# PIP_2_ alters of Ca^2+^ currents in acutely dissociated supraoptic oxytocin neurons

**DOI:** 10.14814/phy2.14198

**Published:** 2019-08-23

**Authors:** Matthew K. Kirchner, William E. Armstrong, Dongxu Guan, Yoichi Ueta, Robert C. Foehring

**Affiliations:** ^1^ Department of Anatomy and Physiology University of Tennessee Health Science Center Memphis Tennessee; ^2^ Department of Physiology, School of Medicine University of Occupational and Environmental Health Kitakyushu Japan

**Keywords:** Ca^2+^ channels, oxytocin, PIP_2_, supraoptic nucleus

## Abstract

Magnocellular neurosecretory cells (MNCs) occupying the supraoptic nucleus (SON) contain voltage‐gated Ca^2+^ channels that provide Ca^2+^ for triggering vesicle release, initiating signaling pathways, and activating channels, such as the potassium channels underlying the afterhyperpolarization (AHP). Phosphotidylinositol 4,5‐bisphosphate (PIP_2_) is a phospholipid membrane component that has been previously shown to modulate Ca^2+^ channels, including in the SON in our previous work. In this study, we further investigated the ways in which PIP_2_ modulates these channels, and for the first time show how PIP_2_ modulates Ca_V_ channel currents in native membranes. Using whole cell patch clamp of genetically labeled dissociated neurons, we demonstrate that PIP_2_ depletion via wortmannin (0.5 *μ*mol/L) inhibits Ca^2+^ channel currents in OT but not VP neurons. Additionally, it hyperpolarizes voltage‐dependent activation of the channels by ~5 mV while leaving the slope of activation unchanged, properties unaffected in VP neurons. We also identified key differences in baseline currents between the cell types, wherein VP whole cell Ca^2+^ currents display more inactivation and shorter deactivation time constants. Wortmannin accelerates inactivation of Ca^2+^ channels in OT neurons, which we show to be mostly an effect on N‐type Ca^2+^ channels. Finally, we demonstrate that wortmannin prevents prepulse‐induced facilitation of peak Ca^2+^ channel currents. We conclude that PIP_2_ is a modulator that enhances current through N‐type channels. This has implications for the afterhyperpolarization (AHP) of OT neurons, as previous work from our laboratory demonstrated the AHP is inhibited by wortmannin, and that its primary activation is from intracellular Ca^2+^ contributed by N‐type channels.

## Introduction

Voltage‐gated Ca^2+^ (Ca_V_) channels regulate transmembrane Ca^2+^ influx in neurons throughout the brain by opening in response to membrane depolarization. Elevated Ca^2+^ as a result of this influx activates neurotransmission, second messenger pathways, other ion channels, and gene expression (Catterall 2011). There are five types of Ca^2+^ currents described in neurons, named L‐, N‐, P/Q‐, R‐, and T‐type. These currents are generated by channels containing α‐subunits named Ca_V_1.1‐1.4 (L‐type), Ca_V_2.1 (P/Q‐type), Ca_V_2.2 (N‐type), Ca_V_2.3 (R‐Type), and Ca_V_3.1‐3.3 (T‐Type) (Simms and Zamponi 2014; Campiglio and Flucher 2015; Dolphin 2018). The channels can be divided into high‐voltage (HVA) and low‐voltage activated (LVA) channels based on their activation voltage dependence. T‐type channels are the only members of the LVA group while the other four make up the HVA group. In addition to their voltage dependence, these channels are modulated by an array of mechanisms including auxiliary *β*‐subunits, calmodulin, G‐proteins, and intracellular Ca^2+^.

The plasma membrane‐bound phospholipid phosphatidylinositol 4,5 bisphosphate (PIP_2_) regulates both K_V_ and Ca_V_ channels (Suh et al. 2010; Hille et al. 2015). PIP_2_ is a long‐chain phospholipid found on the inner leaflet of cell membranes and it is critically involved in the maintenance of the IP_3_/DAG second messenger pathway. PIP_2_ is generated and broken down in the cell membrane. This production is cyclical, and results in a basal production of PIP_2_ regardless of a cell’s current PIP_2_ demand (Xu et al. 2003). In addition to the IP_3_/DAG pathway, PIP_2_ controls a wide range of cellular functions including organization of filamentous actin, cellular differentiation, exocytosis, and ion channel maintenance (Eberhard et al. 1990; Sun et al. 1999; Hilgemann et al. 2001). PIP_2_’s modulation of ion channels is well known. KCNQ (K_V_7) channels are the best documented example (Li et al. 2005; Suh et al. 2006; Kim et al. 2017a), but evidence also exists for modulation of heterologously expressed Ca_V_ channels by PIP_2_. TsA201 cell lines transfected with different Ca_V_ channels show drastically reduced L‐ and N‐type currents acceleration of their inactivation after PIP_2_ depletion (Suh et al. 2010). Retarding of time‐dependent run‐down of P/Q‐type current occurs when excess PIP_2_ is supplied to the cells (Wu et al. 2002).

Magnocellular neurosecretory cells (MNCs) are large (~25 *μ*m) neurons that secrete oxytocin (OT) or vasopressin (VP) via action potential‐triggered exocytosis, and which express L‐, N‐, P/Q‐, R, and T‐type voltage‐dependent Ca^2+^currents (Poulain and Wakerley 1982; Foehring and Armstrong 1996; Fisher and Bourque 1996). Our interest in PIP_2_ modulation of Ca_V_ channels is spurred by previous work of our group on PIP_2_’s modulation of Ca^2+^‐dependent afterhyperpolarizations (AHPs) in these neurons. We determined that PIP_2_ was required for AHP generation in OT, but not VP neurons (Kirchner et al. 2017). Our previous study suggested that PIP_2_ regulates AHP generation by modulating Ca_V_ channels in OT neurons but did not identify which Ca^2+^ channels were affected or by what mechanisms. Furthermore, we found that both medium (mAHP) and slow (sAHP) Ca^2+^‐dependent afterhyperpolarizations (AHPs) are coupled tightly to N‐type channel currents in OT neurons, whereas in VP neurons the mAHP is coupled to N‐type channels and the sAHP is coupled to R‐type channels (Kirchner et al. 2018). Given the numerous observations of PIP_2_/N‐type interactions in previous work (Wu et al. 2002; Delmas and Brown 2005; Michailidis et al. 2007), and the dependence of MNC AHPs on PIP_2_ and N‐currents, we tested for the modulation of whole cell and N‐type Ca^2+^ currents by PIP_2_ in acutely dissociated MNCs from transgenic Wistar rats expressing either red fluorescent or green fluorescent protein in OT or VP neurons, respectively. To our knowledge, this work provides the first direct evidence for PIP_2_ modulation of specific Ca_V_ channels in native neurons.

## Methods

### Ethical approval and animals

These studies were performed on transgenic female Wistar rats containing the AVP‐eGFP fusion and/or the OT‐mRFP1 fusion transgenes (Ueta et al. 2005; Katoh et al. 2011). These rats weighed between 140 and 230 g and aged 6–12 weeks old. The UTHSC IACUC review board approved all experiments and the experiments conform to the principles of UK regulations as described in (Drummond 2009). Animals were on an ad libitum diet. For use in experiments, rats were deeply anesthetized with ketamine/xylazine (10% xylazine; 100 mg kg^−1^), perfused through the heart with artificial cerebrospinal fluid (aCSF) with NaCl replaced by 210 mmol/L sucrose, then decapitated via guillotine. The brains were then removed and sliced for use in whole cell patch clamp electrophysiology. The study was performed only on female rats since the primary focus of the laboratory was plastic changes that occur in these neurons throughout the reproductive cycle (Stern and Armstrong 1996; Teruyama and Armstrong, 2002, 2005; Wang et al. 2018).

### Acutely dissociated neuron preparation

**Figure 1:**
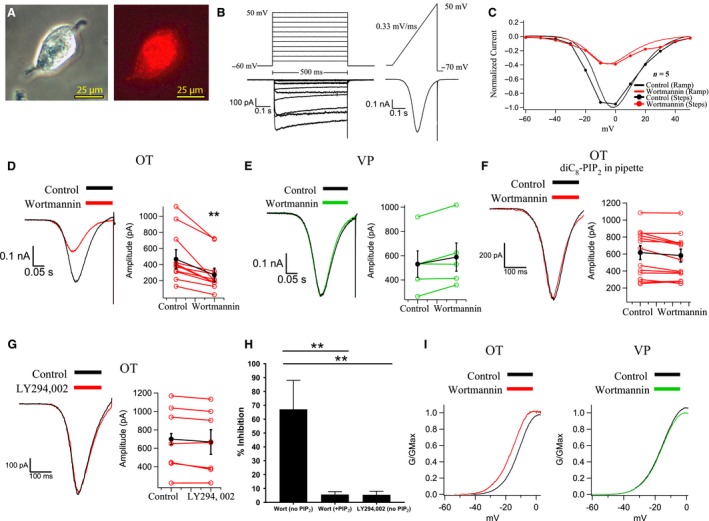
Wortmannin depletion of PIP_2_ inhibits whole cell Ca^2+^ currents in OT neurons but not VP neurons. (A) (*left*), SON neuron visualized with differential interference optics or (*right*) green light excitation (510–560 nm) filter, revealing the OT‐mRFP label. (B) To test whether the I–V relationship of whole cell Ca^2+^ currents generated from ramps reflected the currents seen with steps to specific voltages, we plotted I–V curves from OT neurons (*n* = 5) from both steps (*upper left*) and ramps (*upper right*). The step protocol consisted of a family of steps from a holding potential of −60 mV to +50 mV in 10 mV increments for 500 msec before returning to baseline. (*lower left*) Current traces in the same cell in response to the step protocol. The ramp protocol ramped from −60 mV to +50 mV at a rate of 0.33 mV/msec. (*lower right*) Currents in response to the ramp protocol. (C) Summary plot of averaged currents from normalized ramps and steps in OT neurons before and after wortmannin (*n* = 5 cells). All traces are normalized to the control trace. Note the similarity in the I–V relationships between the two protocols under control conditions and similar effects of wortmannin with both voltage protocols. (D) (*left*) Whole cell Ca^2+^ currents generated *via* ramp protocol from an mRFP1‐labeled OT neuron before and after wortmannin (0.5 *µ*mol/L). (*right*) Summary graph of wortmannin’s effect on OT neurons. Wortmannin inhibited whole cell Ca^2+^ currents by 52.2 ± 8% (*n* = 12, **P < 0.01). (E) (*left*) Whole cell Ca^2+^ currents generated *via* ramp protocol from a GFP‐labeled VP neuron before and after wortmannin (0.5 *µ*mol/L). (*right*) Summary graph of wortmannin’s effect on VP neurons. Wortmannin had no significant effect on whole cell VP Ca^2+^ currents (*n* = 5, *P* > 0.5). (F) Same experiment as Fig. 1D except with diC_8_‐PIP_2_ (30 *µ*mol/L) supplemented in the internal solution. (*left*) Whole cell Ca^2+^ currents generated via ramp protocol from an mRFP1‐labeled OT neuron before and after wortmannin. (*right*) Summary graph of wortmannin’s effect on OT neurons in the presence of diC_8_‐PIP_2_. Wortmannin had no significant effect on whole cell OT Ca^2+^ currents when diC_8_‐PIP_2_ was dialyzed through the pipette (*n* = 13, *P* > 0.6). (G) (*left*) Whole cell Ca^2+^ currents generated via ramp protocol from an OT neuron before and after application of the PI3K inhibitor LY294, 002. (*right*) Summary graph of LY294, 002’s effect on OT neurons. LY294,002 had no significant effect on whole cell OT Ca^2+^ currents (*n* = 8, *P* > 0.6). (H) Summary chart comparing the % inhibition in response to respective drug applications. Cells without diC_8_‐PIP supplementation demonstrated significantly reduced current after wortmannin compared to cells that had diC_8_‐PIP dialysis or LY294,002 application (**P < 0.001). (I) Steady‐state activation plots for OT (*left*; *n* = 6) and VP (*right*; *n* = 6) neurons. These curves represent averages of neurons derived from ramp protocols. While neither cell type demonstrates changes in slope, after wortmannin OT neurons demonstrate a significant hyperpolarizing shift of −4.6 mV measured at the half activation voltage (*V*_1/2_).

After removal, the brain was placed under a dissecting microscope for the dissection of supraoptic tissue. The brain was placed with the ventral surface up. Using iris scissors, a horizontal strip of tissue was excised from the ventral surface of the brain containing the supraoptic nucleus attached to the optic chiasm/tract. This strip of tissue was divided into four even strips of optic tract with hypothalamus attached. These explants were then submerged in aCSF bubbled with 95% O^2^‐5% CO^2^. After resting for a minimum of 30 min, tissue was transferred to a glass chamber warmed by a water bath at 34 °C. The chamber containing the tissue was aCSF + 1 mg/mL of *Streptomyces griseus* Type XIV enzyme (Sigma‐Aldrich) bubbled with 95% O^2^‐5% CO^2^ for 24–30 min. After enzymatic treatment, tissue was transferred to a glass test tube containing a solution (in mmol/L): 140 Na^+^ Isethionate, 2 KCl, 0.1 CaCl_2_, 4 MgCl_2_, 20 Glucose, 10 HEPES, pH balanced to 7.2 with NaOH. The tissue was then titrated through flame‐polished glass pipettes three times in successfully smaller diameter pipettes. The supernatant was removed and plated onto a culture dish, which was placed into the chamber of an inverted microscope (Nikon Diaphot 300). The cells were allowed 7 min to settle on the dish. A background flow of Hanks buffered saline solution (HBSS) was administered and cells were visualized (Fig. 1A). Imaging occurred on the microscope with DIC and fluorescence with two filter blocks for excitation of either AVP‐eGFP (470–490 nm) or OT‐mRFP1 (510–560 nm).

### Electrophysiology

Recording pipettes (2.5–5 MΩ) were pulled from borosilicate glass with an outer diameter of 1.5 mm using a P‐1000 Flaming/Brown horizontal micropipette puller (Sutter Instruments, Sovato, CA). The pipettes were coated to near the tip in bee’s wax to minimize capacitive artifacts during recordings. The pipette internal solution consisted of (in mmol/L): 180 N‐methyl‐d‐glucamine, 4 MgCl_2_, 40 HEPES, 2 Na‐ATP, 0.4 Mg‐GTP, 12 phosphocreatine, 0.1 leupeptin, 10 EGTA; pH 7.2 adjusted with TEA‐OH; 270–280 mOsmol (kg H_2_O)^−1^. Cells were perfused with a background solution of HBSS. After achieving a whole cell patch, a Ca^2+^ current‐isolating solution was delivered to the cells *via* a gravity‐driven multibarrel array of glass capillary tubing (150 *µ*m OD) mounted on a manipulator planted opposite to the recording pipette. Ca^2+^ currents were isolated with a solution in which Ba^2+^ replaced Ca^2+^ as the charge carrier. This was done because Ba^2+^ produces larger currents compared to Ca^2+^, it blocks K^+^ channels, and it minimizes the activation of intracellular Ca^2+^‐dependent mechanisms. This solution contained (in mmol/L): 10 Glucose, 10 HEPES, 5 BaCl_2_, 150 TEA‐Cl, pH 7.2 balanced with TEA‐OH. Drugs were administered using this same multibarrel array. Pharmacological agents included wortmannin (0.5 *µ*mol/L; Tocris Biosciences) to deplete PIP_2_, the L‐type blocker nifedipine (Nif, 5 *µ*mol/L; Sigma‐Aldrich), the N‐type blocker ω‐conotoxin GVIA (CnTx GVIA, 1 *µ*mol/L; Alomone Labs), the P/Q‐type blocker agatoxin IVA (AgTx‐IVA, 0.5 *µ*mol/L; Alomone Labs), and the R‐type blocker SNX‐482 (0.3 *µ*mol/L; Alomone Labs). Some experiments were performed with water‐soluble synthetic diC_8_‐PIP_2_ in the pipette (30 *µ*mol/L; Echelon Biosciences Inc.). Recordings were filtered at 2 kHz. Series resistance was corrected online by 60–85%.

**Figure 2:**
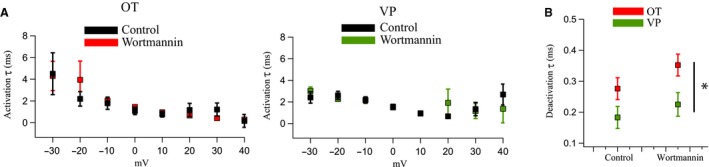
Wortmannin does not significantly affect activation or deactivation time constants in OT and VP neurons. (A) Measurements of activation time constants (*τ*) determined from Ca^2+^ currents generated *via* a step protocol from a holding potential of −60 mV to potentials between −30 mV and +40 mV in OT (*left*) and VP (*right*) neurons. Activation of the current was well fit by a single exponential function (first order). Activation *τ* was not significantly altered in either cell type after wortmannin application (*P* > 0.05). (B) We fit tail currents to a double exponential function, interpreting the first, faster *τ* value as the deactivation *τ*. Deactivation τ was unaffected by wortmannin (*P* > 0.05), but there was a significant difference between OT and VP neurons (*P < 0.05) (Two‐way repeated measures ANOVA).

All recordings in this study were obtained in voltage clamp. We used a ramp protocol at 0.33 mV/ms to evoke isolated Ca^2+^ currents at voltages from −60 mv to +50 mV. We also used a step protocol to evaluate time constants of activation, inactivation, and tail currents (a family of steps from −60 mV to +50 mV in 10 mV increments, 500 msec step duration,) (Fig. 2). All neurons were also tested in 400 *µ*mol/L extracellular Cd^2+^ to confirm measurement of Ca^2+^ currents at the end of the recording. Cells whose series resistance exceeded 20 MΩ or changed more than 20% during the duration of the recording were excluded from analysis.

**Figure 3:**
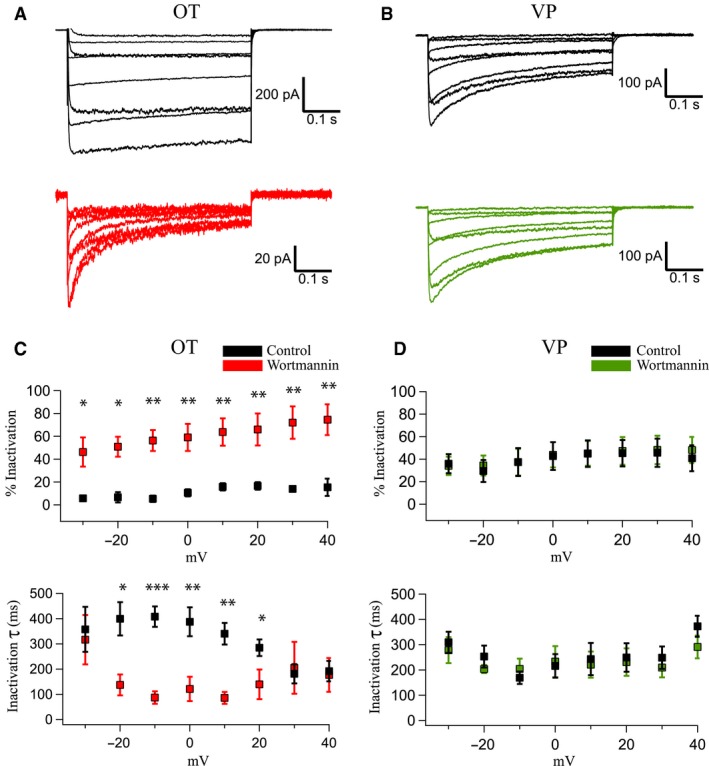
Inactivation properties of OT and VP neurons before and after wortmannin. (A) Ca^2+^ currents in response to voltage steps from a holding potential of −60 mV to +40 mV in OT cells before (*top*) and after (*bottom*) wortmannin application. Note the difference in scale for the wortmannin currents. We expanded the scale of the wortmannin traces because the greatly decreased current amplitude makes it difficult to see the inactivation changes at the original scale. (B) Ca^2+^ currents in response to voltage steps from a holding potential of −60 mV from −30 mV to +40 mV in a VP cell before (*top*) and after (*bottom*) wortmannin application. The inactivation time course in VP neurons was faster compared to their OT counterparts in controls, but slower and less complete compared to OT neurons after wortmannin. (C and D) We compared wortmannin’s (0.5 *µ*mol/L) effect on % Inactivation and inactivation time constants of Ca^2+^ currents in OT (C) and VP (D) neurons from a holding potential of −60 mV to a step of −30 mV to +40 mV. There were significant differences in baseline % Inactivation and inactivation time constants of the currents between OT and VP neurons at holding potentials between ‐10 and +30 mV (*P < 0.05, comparison not explicitly shown). OT neurons revealed significantly increased % Inactivation and faster time constants after wortmannin. Note the lack of wortmannin modulation in VP cells. Significance values are marked with asterisks as follows: *P < 0.05, **P < 0.01, and ***P < 0.001. % Inactivation was measured as: ((Peak‐Steady State)/Peak) × 100.

**Figure 4:**
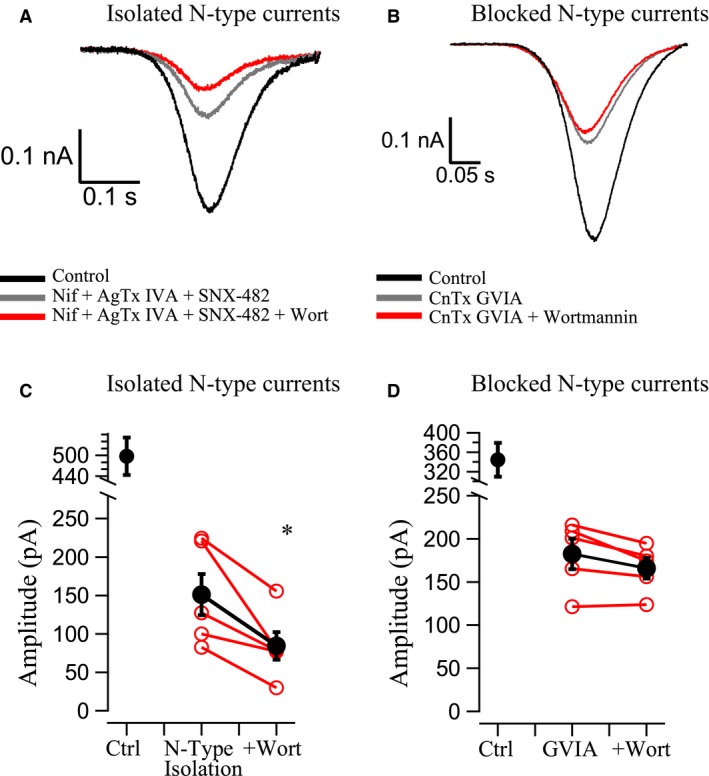
PIP_2_ significantly reduces pharmacologically isolated N‐type Ca^2+^ currents in OT neurons. PIP_2_ depletion *via* wortmannin (0.5 *µ*mol/L) inhibited isolated N‐type currents (A and C) while unaffecting the Ca^2+^ channel currents remaining after N‐type channels were blocked with CnTx GVIA (1 *µ*mol/L) (B and D). (A) Ca^2+^ channel current from a neuron (*black trace*) where N‐type channels were isolated by blocking the other HVA Ca^2+^ channels (*gray trace*) with L‐type blocker nifedipine (Nif, 5 *µ*mol/L), P/Q‐type blocker agatoxin IVA (AgTx‐IVA, 0.5 *µ*mol/L), and R‐type blocker SNX‐482 (0.3 *µ*mol/L). The neurons were subsequently treated with wortmannin (*red trace*), which reduced the isolated N‐type current. (B) Ca^2+^ channel current from a neuron (*black trace*) where N‐type channels were blocked with conotoxin GVIA (CnTx GVIA, 1 *µ*mol/L) (*gray trace*). The neurons were subsequently treated with wortmannin (*red trace*) to evaluate PIP_2_ depletion effects on the remaining HVA Ca^2+^ channel currents. (C) Summary data of peak Ca^2+^ channel currents from ramps. Isolation of N‐type currents resulted in a 69.6 ± 6% reduction in whole cell current. Wortmannin significantly inhibited the isolated N‐type current by 44 ± 6% (*P < 0.05). Red traces represent individual neurons while the black trace is the mean ± SEM. (D) Summary data of peak Ca^2+^ channel currents from ramps. Block of N‐type currents resulted in a 45.6 ± 6% reduction in whole cell current. Wortmannin slightly inhibited the current remaining after GVIA by 12 ± 1%, but this was not statistically significant (*P* > 0.05). Red traces represent individual neurons while the black trace is the average.

**Figure 5:**
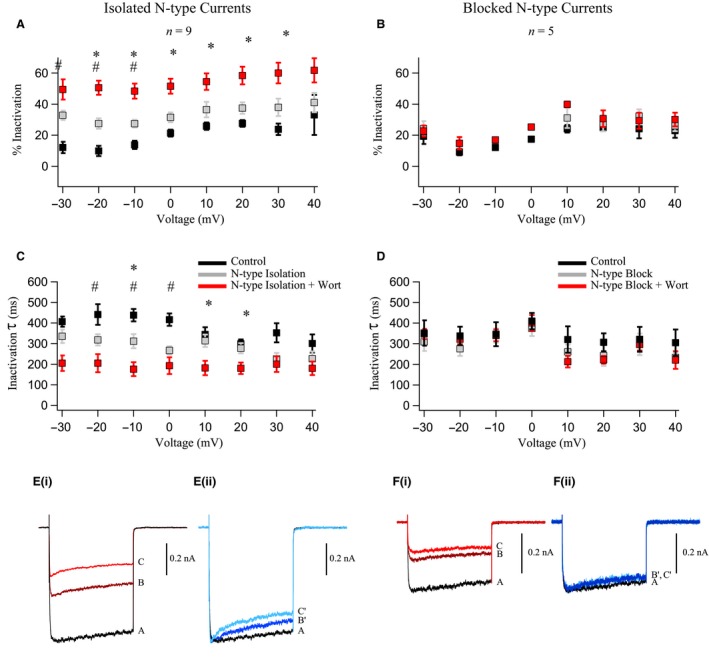
Wortmannin significantly reduced % Inactivation and accelerates inactivation of N‐Type currents in OT neurons. PIP_2_ depletion via wortmannin (0.5 *µ*mol/L) reduced the % Inactivation of the isolated N‐current (A, C and E) but not Ca^2+^ currents remaining after N‐type channels were blocked (B, D and F) in OT neurons. Significant differences between control and N‐type isolation are marked with a pound symbol (^#^ *P* < 0.05) while an asterisk (*P < 0.05) marks the significant difference between N‐type currents before and after wortmannin. (A) Summary data of % Inactivation under control conditions (*black points*), N‐type channel isolation (*gray points*), and N‐type isolation + wortmannin (*red points*). (B) Summary data of % Inactivation under control conditions (*black points*), N‐type channel block (*gray points*), and N‐type block + wortmannin (*red points*). No significant differences occurred between any groups (*P* > 0.05). (C) Summary data of inactivation time constants under control conditions (*black points*), N‐type channel isolation (*gray points*), and N‐type isolation + wortmannin (*red points*). (D) Summary data of inactivation time constants under control conditions (*black points*), N‐type channel block (*gray points*), and N‐type block + wortmannin (*red points*). No significant differences occurred between any groups (*P* > 0.05). (Ei) Representative traces of Ca^2+^ channel currents generated by a 500 msec voltage step to −10 mV from a holding potential of −60 mV. Trace A is a baseline Ca^2+^ channel current, trace B is the pharmacologically isolated N‐type current, and trace C is the isolated N‐type current after wortmannin. (Eii) Traces B′ and C′ are the same respective traces as in Ei, except scaled to the peak of the control to highlight the effect of PIP_2_ depletion on % Inactivation independent of the reduction in current. (Fi) Representative traces of Ca^2+^ channel currents generated by a 500 msec voltage step to −10 mV. Trace A is a baseline Ca^2+^ channel current. Trace B is the whole cell Ca^2+^ channel current with blocked N‐type current. Trace C is the current after subsequent addition of wortmannin. (Fii) Traces B′ and C′ are the same respective traces scaled to the peak of the control. In contrast to (E), wortmannin did not significantly change the % Inactivation when N‐type current was blocked.

**Figure 6:**
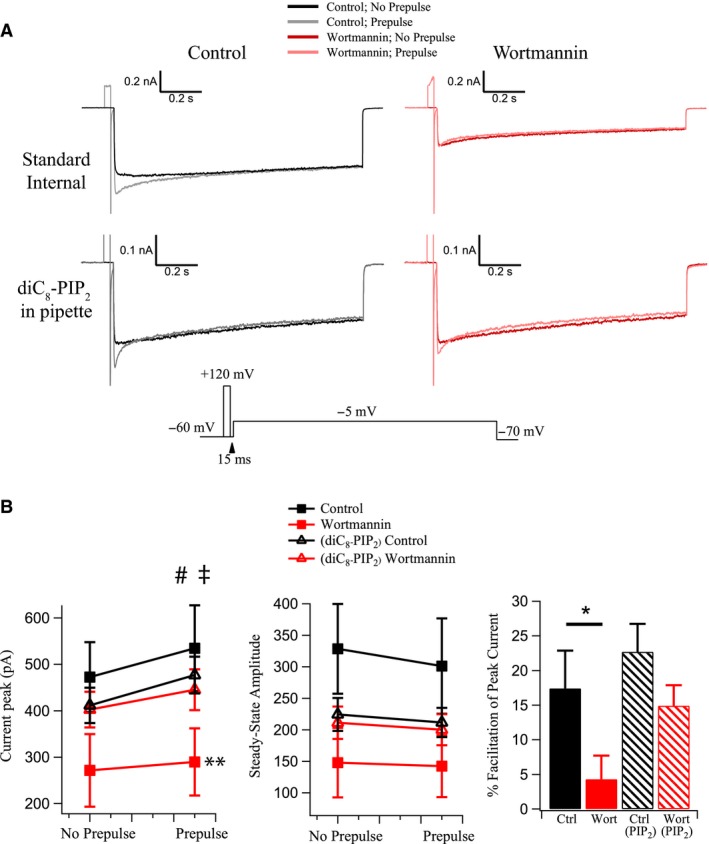
PIP_2_ is necessary for voltage‐dependent facilitation of Ca^2+^ current on OT neurons. We tested the effects of PIP_2_ depletion on facilitation of Ca^2+^ channel currents by a depolarizing prepulse (c.f., Bean 1989). Ca^2+^ currents were evoked with a step to from −60 mV to −5 mV in OT neurons. Current was then evoked by the same test pulse but following a 30‐msec prepulse to +120 mV. We performed this two‐step protocol under control conditions (*left*) and in the presence of wortmannin (*right*), with (*upper*) and without (*lower*) diC_8_‐PIP_2_ in the pipette. (A) Example of Ca^2+^ currents with and without the prepulse, before and after wortmannin with the corresponding voltage protocol provided (*bottom*). (B) Summary data for the effects of prepulse, wortmannin, and diC_8_‐PIP_2_ in the pipette on the current peak (pA) and % facilitation. (*left*) Wortmannin inhibited peak Ca^2+^ channel currents when diC_8_‐PIP_2_ was absent (**P < 0.01). Inclusion of a prepulse significantly increased the peak current whether diC_8_‐PIP_2_ was absent (^#^ *P* < 0.05) or present (^ǂ^ *P* < 0.05) in the internal solution. (*middle*) Neither wortmannin nor diC8‐PIP_2_ affected the steady‐state amplitude of currents before and after a prepulse. (*right*) Histogram depicts % facilitation caused by the prepulse. Wortmannin significantly prevents the facilitation in standard solution (*P < 0.05). diC_8_‐PIP_2_ in the pipette prevents wortmannin’s inhibition of the facilitation.

To evaluate statistical differences between groups, we primarily used a two‐way repeated measures ANOVA to evaluate the differences between OT and VP neurons before and after drug application (Figs. 1, 2, 3). We also used a repeated measures ANOVA to analyze wortmannin’s effect on isolated and blocked N‐type channels (Figs. 4 and 5), to analyze the prepulse facilitation data before and after wortmannin, and for comparing groups with and without diC_8_‐PIP_2_ in the pipette (Fig. 6). We used a Sidak test for all *post hoc* analyses. All statistics were performed in SPSS (IBM).

## Results

### PIP_2_ depletion inhibits isolated Ca^2+^ currents in OT neurons

We previously demonstrated that PIP_2_ depletion via wortmannin inhibited whole cell Ca^2+^ currents in slices from wild‐type Sprague–Dawley rats (OT neurons) (Kirchner et al. 2017), and that N‐type channels were most closely coupled to AHPs, especially in OT neurons (Kirchner et al. 2018). Here we examined the nature of this modulation in acutely dissociated MNCs, a preparation where space clamp artifacts are greatly minimized. Wortmannin inhibits both PI4K*α*, and PI3K, enzymes that act as the rate‐limiting steps for PIP_2_ and PIP_3_ production, respectively (Nakanishi et al. 1995) and effectively depleting PIP_2_ from the neurons. Here we tested wortmannin’s effects under conditions where voltage was better controlled and solutions could be rapidly changed using dissociated neurons from transgenic Wistar rats.

To determine the consistency between ramp and step protocol I–V relationships, we plotted data from both protocols together (Fig. 1B). The traces obtained from all cells were averaged and then normalized to the maximal current. We first did two comparisons: control steps versus control ramps and wortmannin steps versus wortmannin ramps (Fig. 1C). We determined that the ramp protocol provides an I–V curve that accurately reflects one plotted from peak currents generated in the step protocol in each case.

We observed the following time course for wortmannin effects on Ca_V_ currents: Cells responded to wortmannin approximately 10–30 sec after drug delivery was initiated. An initial fast stage of inhibition occurred over 1–5 sec and was followed by the Ca^2+^ current settling into steady‐state by 30 sec. Measurements of the drug effects were taken during this steady‐state.

Wortmannin (1 *µ*mol/L) inhibited ramp‐generated Ca^2+^ current amplitudes by 52.2 ± 8% in OT neurons (*n* = 12; *P* < 0.001; Fig. 1D). Consistent with our previous observations in slices (Kirchner et al. 2017), wortmannin failed to inhibit ramp‐generated Ca^2+^ currents in VP neurons (*n* = 13; *P* > 0.4; Fig. 1E). The wortmannin effect was prevented when the water soluble, synthetic PIP_2_ analog dic_8_‐PIP_2_ (30 *µ*mol/L) was supplied to the cells via the patch pipette (*n* = 5; *P* > 0.5; Fig. 1F). Because wortmannin inhibits PIP_3_ production (via PI3K) in addition to PIP2 production (via PI4K*α*), we tested the highly specific PI3K blocker, LY294,002 on the Ca^2+^ currents. LY294,002 produced no significant inhibition of the current (*n* = 8; *P* > 0.3; Fig. 1G). This experiment increased confidence that wortmannin is inhibiting Ca^2+^ currents by depleting PIP_2_. Wortmannin also inhibited a much larger percentage of current compared to wortmannin with diC_8_‐PIP_2_ present the patch pipette, and compared to LY294,002 (*P* < 0.01; Fig. 1H).

In addition to an effect of PIP_2_ depletion on current amplitude, we observed a leftward shift in the voltage‐dependent activation for OT neurons (Fig. 1I). To determine the steady‐state voltage dependence of activation, we generated a plot from ramps wherein currents were converted to conductance (*G*): $G=\frac{I}{\left(V_{\text{step}}-V_{\text{rev}}\right)}$. Every point from the ramp‐generated current was calculated to G/GMax, using a Boltzmann equation and plotted versus voltage (Fig. 3E). The Boltzmann equation used was:

$$G/G_{\max}=\frac{1}{1+e^{V-V_{0.5}/k}}$$

*V*_0.5_ represents half activation and *k* represents the slope factor. Wortmannin negatively shifted *V*_0.5_ for activation by −4.6 mV (*n* = 5; *P* < 0.05). There was no change in the slope of the curve (control 6.14 ± 0.01 vs. wortmannin 5.94 ± 0.01; *P* > 0.5). VP neurons demonstrated no changes in *V*_0.5_ (control −15.8 vs. wortmannin −16.1) or slope (control 6.34 ± 0.01 vs. wortmannin 6.10 ± 0.01) (Fig. 1I).

### PIP_2_ effects on Ca^2+^ channel gating

We generated Ca^2+^ currents using a step protocol to measure the kinetics of current activation, deactivation, and inactivation as a function of membrane voltage. At all membrane potentials measured, wortmannin had no significant effect on activation τ in either OT or VP neurons (*n* = 5) (Fig. 2A). Wortmannin also had no significant effect on the deactivation τ measured from tail currents at −70 mV (*P* > 0.05). However, in control solution, the deactivation τ was significantly slower in OT versus VP neurons (*P* < 0.05, Fig. 2B).

We next measured both percent inactivation (% Inactivation) and the inactivation time constants for Ca^2+^ channel currents. We operationally defined % Inactivation as the percent change in current between peak and steady‐state values. We used this measure in addition to time constants because in these cells, Ca^2+^ channel currents do not fully inactivate during steps, even up to 5 sec duration. This is particularly relevant to OT neurons. As a result, time constant measurements in OT neurons under control conditions are likely underestimated, but we report them because the kinetics of inactivation clearly change significantly after wortmannin. Because longer step durations increased current run‐down over time, we opted to use a 500 msec step and measure the time constant as well as % Inactivation within this step. The time constant was measured using a monoexponential fit.

We observed significant increases in % Inactivation and inactivation time constants in OT neurons in the presence of wortmannin at potentials ranging from −30 to +40 mV (10 mV increments) (*P* < 0.05, 0.01; Fig. 3A and C). Some steady‐state current remained after wortmannin (Fig. 5E), which we measured at the end of the pulse. VP neurons were unaffected by wortmannin application in this regard, at any potential (Fig. 3B and D). Additionally, VP neurons displayed significantly higher baseline % Inactivation and inactivation time constants compared to their OT counterparts (*P* < 0.05; Data not plotted). This baseline difference is apparent in the control traces of Fig. 3A and B.

### Modulation of N‐type current in OT neurons

As noted earlier, there is a strong precedence for modulation and even the necessity of PIP_2_ for N‐type channel activation using expression systems (Michailidis et al. 2007; Suh et al. 2010). In previous work, we observed that specific Ca^2+^ channel types couple to each AHP component, and specifically, that N‐type channels couple to AHPs in OT neurons (Kirchner et al. 2018). We therefore isolated N‐type currents by using a cocktail of 5 *µ*mol/L Nif (blocks L‐type), 0.5 *µ*mol/L AgTx‐IVA (blocks P/Q‐type), and 0.3 *µ*mol/L SNX‐482 (blocks R‐type). Ca^2+^ channel currents were generated from ramps and application of Nif + AgTx‐IVA + SNX‐482 resulted in a 69.6 ± 6% reduction of the whole cell peak current (Fig. 4A and C). We interpret this remaining current as the isolated N‐type current. Wortmannin inhibited this isolated N‐type by 44 ± 6% (*n* = 5; *P* < 0.05).

We also did the complementary experiment, testing wortmannin’s ability to affect non‐N–type Ca^2+^ currents after N‐type block with 1 *µ*mol/L CnTx GVIA. CnTx GVIA resulted in 46 ± 6% reduction of whole cell current, but wortmannin did not significantly inhibit the CnTx GVIA‐treated current (*n* = 5; *P* > 0.05) (Fig. 4B and D).

Because wortmannin increased the inactivation of whole cell Ca^2+^ currents, we evaluated how much of this inactivation could be attributed to effects at N‐type versus all other HVA currents (Fig. 5). We found that N‐type isolation resulted in a significant reduction in the % Inactivation at −30, −20, and −10 mV (*n* = 7; *P* < 0.05; Fig. 5A). Application of wortmannin to isolated N‐type currents resulted in significant inhibition of % Inactivation at almost all measured potentials (*n* = 7; *P* < 0.05; Fig. 5A). When measuring inactivation time constants, isolation of N‐type current resulted in significant reductions at −20, −10, and 0 mV (*n* = 7; *P* < 0.05; Fig. 5C). Application of wortmannin to the isolated N‐type currents shortened time constants significantly at potentials of −10, +10, and +20 mV (*n* = 7; *P* < 0.05; Fig. 5C). This is most apparent when the isolated N‐type current and the wortmannin‐treated current were scaled to the control amplitude, where rapid inactivation was apparent (Fig. 5Eii). Significance at individual voltages was determined with a Sidak post hoc test.

In contrast to the isolated N‐type currents, we observed no significant changes for % Inactivation or inactivation time constants measured at any potential from the currents remaining after N‐type channels were blocked (*n* = 5; *P* > 0.05; Fig. 5B and D). Again, this is apparent when the traces with CnTx GVIA and wortmannin were scaled to the control amplitude (Fig. 5Fii).

### PIP_2_ and facilitation of Ca^2+^ channels

To further elucidate voltage‐dependent mechanisms of PIP_2_ modulation, we explored the possibility of its involvement in Ca^2+^ channel facilitation. Facilitation in this context is classically defined as an increased Ca^2+^ current following application of a brief, strong positive prepulse (to +120 mV) (Bean 1989). In previous studies, such a prepulse has been shown to reverse G protein‐mediated neurotransmitter modulation to various degrees (Aosaki and Kasai 1989; Bean 1989; Foehring 1996; Colecraft et al. 2000; Stewart and Foehring 2000; Schober et al. 2010). While such a prepulse is not physiological, it is a vital method in testing potential allosteric modulation by PIP_2_. To test whether PIP_2_ affects facilitation, we generated Ca^2+^ currents in OT neurons with and without a 30‐ms prepulse to +120 mV from −60 mV and evaluated facilitation before and after wortmannin. Insertion of a prepulse significantly increased the peak Ca^2+^ channel current by 17.4 ± 5% (facilitation: *P* < 0.05; *n* = 5; Fig. 6). Application of wortmannin inhibited the overall peak current (*P* < 0.01). In the presence of wortmannin, the prepulse failed to significantly increase peak current, modulating it by only 4.3 ± 3% (*P* > 0.05; Fig. 6). In other words, facilitation was significantly reduced by wortmannin compared to controls (*n* = 5; *P* < 0.05; Fig. 6). The prepulse did not affect steady‐state current (*P* > 0.05; Fig. 6B).

We executed the same experiment described above with diC_8_‐PIP_2_ in the patch pipette. Under these conditions, the prepulse significantly facilitated peak current amplitude (*P* < 0.05; Fig. 6B), and wortmannin failed to significantly inhibit peak or steady‐state currents, consistent with previous experiments. In diC_8_‐PIP_2_, wortmannin did not significantly alter facilitation of peak current amplitude (diC_8_‐PIP_2_: control 22.7 ± 3% wortmannin 14.9 ± 3%; *P* > 0.1) or steady‐state current (Fig. 6B). These data suggest that facilitation is compromised after PIP_2_ depletion but that excess PIP_2_ has no further effect on facilitation relative to control PIP_2_ levels.

## Discussion

In this study, we pursued three aims: (1) to determine if PIP_2_ modulates Ca^2+^ currents in native MNCs; (2) to determine the mechanisms underlying the effects of PIP_2_ on Ca^2+^ currents; and (3) to determine if PIP_2_ *specifically* modulated the N‐type channel, which we had shown to be a critical source of Ca^2+^ for generation of AHPs in OT neurons of the SON (Kirchner et al. 2018). In this study, we provide the first direct evidence for PIP_2_ modulation of specific Ca_V_ channels in native neurons. Previous work has characterized this relationship meticulously in expression systems (Gamper et al. 2004; Suh et al. 2010; Vivas et al. 2013). Additionally, indirect evidence exists in acutely dissociated bullfrog sympathetic ganglion neurons, where Ca_V_ currents are modulated by type II luteinizing hormone–releasing hormone in the absence of a PLC inhibitor (Wu et al. 2002). Our primary finding is that inhibition of Ca^2+^ current amplitudes after application of wortmannin in acutely dissociated OT (but not VP) neurons of Wistar transgenic rats is blocked when the patch pipette contained diC_8_‐PIP_2_, affirming that wortmannin’s effect on Ca^2+^ channel currents is related to PIP_2_ depletion. Additionally, the specific PI3K blocker LY294,002 failed to inhibit currents. In contrast to OT neurons, wortmannin had no effect on VP neuron currents. These results are consistent with our previous observations in slices of wild‐type Sprague‐Dawley rats (Kirchner et al. 2017). Finally, our results show that the PIP_2_ modulation is primarily exerted on N‐type Ca^2+^ channels.

Interestingly, we also observed a hyperpolarizing shift in the voltage‐dependent activation of whole cell Ca^2+^ currents. This result is consistent with previous reports in which PIP_2_ depletion resulted in a leftward shift of voltage‐dependent activation of N‐type channels (Suh et al. 2010). In contrast, increased PIP_2_ levels resulted in a hyperpolarizing shift in voltage activation of Kv7 channels (Kim et al. 2016). While it is clear that depletion of PIP_2_ has effects on voltage‐dependent activation of Ca^2+^ currents in OT neurons, the direction and amount of this change would not account for dramatic inhibition of Ca^2+^ current we observed with PIP_2_ depletion (i.e., the leftward shift in activation observed with PIP_2_ depletion would tend to increase current at a given voltage). We also found no effect of wortmannin on the time course of activation or deactivation in OT neurons. In contrast, we observed a significant increase in % Inactivation and the rate of inactivation in OT neurons in the presence of wortmannin.

While Ca^2+^ currents in VP neurons were unaffected by wortmannin, under control conditions, VP neurons demonstrated significantly stronger and faster inactivation at depolarized potentials compared to their OT counterparts. In addition, the deactivation *τ* was smaller in VP neurons than OT under basal conditions. This suggests the two cell types differ in Ca^2+^ channel family and/or subunit populations. This hypothesis is backed by previous observations in which the percent of current blocked by HVA Ca^2+^ channel toxins among *unidentified* MNCs (Fisher and Bourque 1995; Foehring and Armstrong 1996) was highly variable. A later study examining the mRNA levels of Ca^2+^ channels in OT versus VP neurons showed that OT neurons contained higher levels of N‐type mRNA (Glasgow et al. 1999). This same study demonstrated that mRNA levels for specific Ca^2+^ channels and their *β* subunits varied greatly across individual cells of the same cell type, providing further explanation for variability observed in the amount of current inhibited by pharmacological Ca_V_ channel blockers. Interestingly, a recent report showed that N‐type channels in VP neuronal terminals contain a *β*_2_ subunit absent in OT neurons (Ortiz‐Miranda et al. 2010). This subunit is known to greatly alter the biophysical properties of the channel, including its inactivation kinetics (Campiglio and Flucher 2015). Although reports indicate *β*_2_ subunit mRNA amount is approximately double in OT neurons (Glasgow et al. 1999), these measurements reflect the mRNA and not actual translation. Furthermore, while the *β*_2_ subunit may not associate with N‐type channels in VP neuronal terminals, it is unknown whether there are *β*_2_/N‐Type interactions in other VP cellular compartments. Indeed, the presence of *β *subunits, including *β*_2_, determine N‐type channel sensitivity to PIP_2_ depletion in expression systems (Suh et al. 2012). Consistent with different Ca^2+^ channel subunit composition in OT versus VP neurons, we found that % Inactivation was significantly higher in VP neurons under control conditions (Fig. 3).

Our earlier work made it clear that the presence of PIP_2_ allowed for more robust Ca^2+^ channel currents involved in the generation of AHPs (Kirchner et al. 2017), and we subsequently determined that N‐type channels were the Ca^2+^ source for OT AHPs (Kirchner et al. 2018). Additionally, PIP_2_ modulation of N‐type channels has been described in other cell types (Suh et al. 2010; Vivas et al. 2013; Kim et al. 2017b). We therefore focused our efforts on the modulation of specific channel types, and found that wortmannin affected isolated N‐type currents, but that no significant inhibition of current occurred after block of N‐type channels. Furthermore, we observed significant increases in inactivation τ and the % Inactivation of isolated N‐type currents after wortmannin, while these were unaffected after GVIA‐blocked N‐type Ca^2+^ currents. These data demonstrate that N‐type channels are the main Ca^2+^ channel target of PIP_2_ modulation in OT neurons.

The inactivation changes following PIP_2_ depletion are small compared to inhibition of the current peak amplitude, and thus seem unlikely to account for the large inhibition of Ca^2+^ currents we observe. This observation is consistent with those in tsA‐201 cells in which there were large depressions of currents after PIP_2_ depletion but relatively small changes in gating (Suh et al. 2010). Suh et al. (2010) suggested that with reduced PIP_2_, fewer channels were available to open. This is similar to Bean (1989) hypothesis that G‐protein interactions shift Ca^2+^ channels into a “reluctant” mode for channel opening and can be converted to “willing” channels by voltage‐dependent removal of G‐protein inhibition (see also Foehring 1996). PIP_2_ may also encourage or be permissive for the opening of N‐type Ca^2+^ channels, as demonstrated by our data on facilitation (Fig. 6). Inclusion of a depolarized prepulse immediately prior to current generation resulted in marked facilitation of the peak current, an effect which is blocked by the application of wortmannin. Excess PIP_2_ (due to inclusion in the pipette) prevents this effect but results in no further increase in current, suggesting that PIP_2_ must be present for facilitation to occur. Wortmannin prevents the facilitation and elevated PIP_2_ levels prevent (or at least reduce) wortmannin’s effect. Thus, it appears PIP_2_ levels are needed for voltage‐dependent facilitation to occur, perhaps through allosteric effects. Future studies will be required to determine the nature of this relationship.

## Conflict of Interest

None declared.
