# Erratum

**DOI:** 10.14814/phy2.14310

**Published:** 2019-12-13

https://doi.org/10.14814/phy2.14198

In Kirchner, Armstrong, Guan, Ueta, and Foehring (2019), the following error was published in the title on page 1.

Article Title

PIP2 alters of Ca2+ currents in acutely dissociated supraoptic oxytocin neurons

There was an ‘of’ included in the title after alters. This should have read:

Article Title

*PIP_2_ alters Ca^2+^ currents in acutely dissociated supraoptic oxytocin neurons.*

The publisher apologizes for this error.
